# Regulatory Mechanisms of Bud Dormancy: Environmental, Hormonal, and Genetic Perspectives

**DOI:** 10.3390/ijms26062517

**Published:** 2025-03-11

**Authors:** Irfan Ali Sabir, Xinglong Hu, Imran Khan, Yonghua Qin

**Affiliations:** 1 Key Laboratory of Biology and Genetic Improvement of Horticultural Crops (South China), Ministry of Agriculture and Rural Affairs, College of Horticulture, South China Agricultural University, Guangzhou 510642, China; 2 Guangdong Provincial Key Laboratory of Postharvest Science of Fruits and Vegetables, College of Horticulture, South China Agricultural University, Guangzhou 510642, China

**Keywords:** dormancy, chilling hours, hormone, environment, mechanism

## Abstract

Dormancy is a vital adaptive strategy in temperate and boreal plants, particularly fruit trees, enabling them to withstand harsh winter conditions and ensure survival and synchronized growth resumption in spring. This review comprehensively examines dormancy, focusing on its physiological, environmental, and molecular mechanisms. Dormancy is characterized by two distinct phases: endodormancy, which is regulated by internal plant signals and requires cold temperatures for release, and ecodormancy, which is influenced by external environmental factors. These stages are intricately linked to seasonal temperature fluctuations and the plant’s ability to synchronize growth cycles, ensuring survival through harsh winters and optimal growth in warmer seasons. The review delves into the role of chilling requirements, temperature thresholds, and hormonal regulation in the dormancy process, highlighting how these factors influence critical growth events such as budbreak, flowering, and fruiting. Plant hormones, including abscisic acid, gibberellins, and cytokinins, regulate dormancy by modulating gene expression and growth activity. Additionally, we explore the historical development of dormancy research, from early observations of chilling requirements to the formulation of the chilling hours model. Considering ongoing climate change, the review examines how rising winter temperatures may disrupt dormancy cycles, potentially affecting the timing of flowering, fruiting, and overall crop productivity. This shift necessitates new strategies for managing dormancy, particularly in regions experiencing inconsistent or insufficient chilling. The review concludes by discussing practical approaches to enhance dormancy release and mitigate the impact of environmental stress on deciduous fruit tree growth, offering insights into improving agricultural practices amidst a changing climate.

## 1. Introduction

Dormancy is a genetically regulated developmental phase in temperate regions that enables deciduous fruit trees to survive adverse climatic conditions, particularly during winter [1]. It is marked by a short halt in development, which may be categorized into two separate phases: endodormancy and ecodormancy. Endodormancy, or ‘true dormancy’, is regulated by internal physiological factors, whereas ecodormancy, or ‘climatic dormancy’, is influenced by external environmental conditions. The accumulation of adequate chilling during winter is essential for breaking endodormancy, while warmer temperatures are required to terminate ecodormancy. In tree fruit species, the timing of bud dormancy is closely aligned with the phenological cycle, where sufficient winter chilling promotes uniform budbreak, flowering, and fruit development [2].

Woody and perennial plants in temperate and boreal climates have evolved to synchronize their growth cycles with seasonal temperature fluctuations. This includes precise timing of bud dormancy, winter rest, and frost resistance. The duration and severity of winter dormancy are crucial factors influencing the length of the growing season and, ultimately, crop productivity. Dormancy effectively halts shoot and root development to protect the plant from adverse conditions [3,4]. Lang (1987) defined dormancy as a developmental phase where plant growth temporarily ceases [5], while Rohde and Bhalerao (2007) described it as the inability of meristems or other growth organs to resume development, even under optimal environmental conditions [6]. Lang et al. (1987) further refined the concept of dormancy by introducing three stages: paradormancy, endodormancy, and ecodormancy. Paradormancy refers to growth inhibition in specific plant tissues, such as those controlled by apical dominance, often due to the production of inhibitory substances. During endodormancy, even under ideal temperature conditions, buds remain dormant because they have not undergone sufficient chilling hours. In contrast, ecodormancy represents the stage when bud development can resume after exposure to relatively brief warm periods [7]. If the chilling requirement is not met during winter, plants may experience irregular or delayed budbreak, reduced branch vigor, limited flowering, and poor fruit development. For perennial fruit trees, global warming presents a significant threat by disrupting the accumulation of chilling hours necessary for optimal budbreak. A lack of adequate chilling directly impacts flower formation, potentially leading to dramatic reductions in fruit production [8]. Warmer winters and more frequent heat waves can lead to disruptions in plant development, including delayed or absent budbreak, prolonged blooming cycles, and diminished flower quality. These climate-induced changes may have serious implications for horticultural productivity and the stability of fruit production systems [9]. During the vegetative growth phase, plants subjected to inadequate chilling may exhibit abnormalities such as stunted branch development, reduced leaf size, and smaller fruits [9]. Therefore, developing strategies to ensure more consistent and timely dormancy release is critical to mitigating the challenges posed by insufficient cold temperatures or excessive warmth during the dormant period.

The study of tree dormancy traces back to the early 19th century. In 1801, Sir Thomas Knight first documented that temperate woody perennials depend on exposure to cold winter temperatures to overcome dormancy and initiate synchronized spring growth, laying the foundation for understanding the chilling requirement in perennial plants [10]. By the mid-twentieth century, much of the foundational knowledge on dormancy had been established [11,12]. During this period, researchers also explored the use of various bioactive substances to overcome the effects of insufficient chilling [11], leading to the development of the “chilling hours” model. This temperature-based model was one of the first to address the impacts of mild winter temperatures on dormancy release [13]. Frederick V. Coville’s pioneering work in the early 20th century involved exposing seedlings, potted trees, and shoots to different temperature regimes, demonstrating that low temperatures break dormancy by converting starches into soluble sugars [13,14]. As winter dormancy in fruit trees becomes an increasingly critical area of study, particularly in the context of climate change, scientists and producers are paying closer attention to its effects. Warming winters may dramatically alter the timing of key phenological events, such as blooming, fruit ripening, and leaf senescence, potentially disrupting the productivity of deciduous fruit tree orchards. Additionally, climate change is altering the suitability of orchard environments for certain tree species and cultivars, prompting growers to adapt by shifting the types of fruit trees planted in key production areas [15,16].

## 2. Hormonal Impact on Bud Dormancy Regulations

Plant hormones, or phytohormones, are small signaling molecules that regulate a wide range of physiological processes, even at low concentrations. They play a crucial role in coordinating growth, development, and responses to environmental stimuli throughout the plant’s life cycle. Moreover, these hormones play a pivotal role in enabling plants to adapt to a wide range of environmental challenges, including both biotic (caused by living organisms) and abiotic (non-living environmental factors) stresses. Plant hormones are typically classified into five major types: gibberellins (GAs), auxins (e.g., indole-3-acetic acid), cytokinins (CTK), ethylene (ET), and abscisic acid (ABA). Additionally, other plant-derived compounds, such as salicylic acid (SA), jasmonic acid (JA), strigolactones (SLs), and brassinosteroids (BR), exert hormone-like effects and contribute to plant regulation.

**Figure 1:**
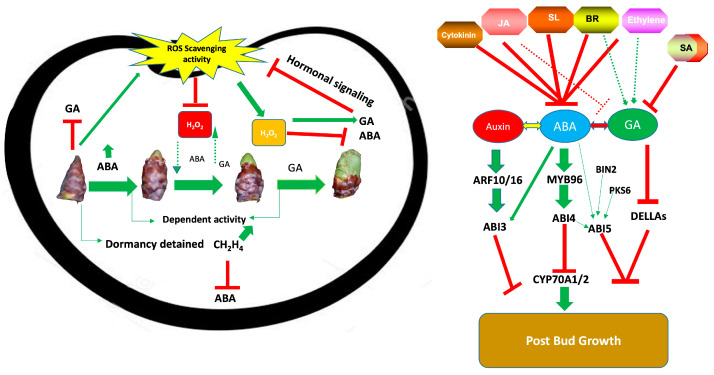
Hormonal orchestration during bud dormancy and dormancy release.

Many of these hormones are known to be involved in the complex regulation of processes like bud dormancy (Figure 1) [17]. Advances in our understanding of hormone signaling have been driven by improvements in molecular and subcellular techniques, particularly in areas such as hormone perception, signal integration, and signal transduction. These developments have highlighted the intricate interactions between different hormones in regulating plant responses. Furthermore, genetic mutagenesis in model plants has provided valuable insights into the roles of specific components involved in hormone synthesis and signal transduction, particularly under conditions that trigger dormancy. Notably, hormones such as ABA, GA, auxin, JA, SA, and reactive oxygen species (ROS) interact with one another to modulate dormancy processes (Figure 1).

Recent advancements in proteomics and bioinformatics, which provide comprehensive insights into key genomic processes at a given time, have greatly enhanced our understanding of hormone regulation, particularly concerning bud dormancy.

## 3. ABA Impact on Bud Dormancy

ABA regulates several aspects of plant growth and development and acts as a vital messenger in response to stress [18]. It is especially vital in controlling organ senescence and abscission, which is considered the primary functions of ABA [18,19]. Moreover, ABA is crucial for regulating dormancy, as dormancy essentially involves the suspension of meristematic growth, requiring a halt in overall plant development [20]. Numerous physiological, genetic, and molecular studies have highlighted the importance of ABA in regulating bud dormancy [2,21,22]. During dormancy establishment, endogenous ABA levels rise, and a decline is observed when dormancy is released. For example, in *Vitis vinifera* (grapevine), the concentration of ABA rises to threefold during the initiation of dormancy and gradually declines as dormancy diminishes. This fluctuation aligns closely with an increase in the budbreak ratio when plants are subjected to controlled or forced environmental conditions [19]. Similar patterns of ABA fluctuation have been observed in other species such as sweet cherry (*Prunus avium*) [23], pear (*Pyrus pyrifolia*) [24], and peach (*Prunus persica*) [25].

It has been suggested that the short-day (SD) photoperiod before dormancy development triggers the rise in ABA content, and that its reduction is linked to chilling accumulation [26]. This correlation between ABA levels and dormancy depth supports ABA’s involvement in both the initiation and progression of dormancy [27]. Further evidence of ABA’s central role in dormancy management comes from studies showing that the reduction of ABA levels in latent buds can enforce dormancy release. For instance, the application of fluridone, an inhibitor of ABA synthesis, to dormant *Rosa hybrida* buds resulted in the production of new leaf primordia. This finding suggests that prolonged in situ ABA synthesis is necessary for the maintenance of bud dormancy. In a similar manner, the catabolism of ABA was effectively triggered in grapevines, and the levels of ABA were decreased when the buds were treated with hydrogen cyanamide (HC), which is a well-known chemical that breaks buds [19,28]. This observation is supported by a transgenic pear study, where dormancy release was accelerated through the overexpression of the ABA catabolism enzyme 8′-hydroxylase (ABA8OH) [27]. These findings reinforce the idea that ABA suppresses primordia growth during dormancy, and that a reduction in ABA levels and de novo ABA production are likely necessary for maintaining bud inactivity even after dormancy is established. The decline in ABA levels is therefore an important factor in dormancy release.

Moreover, research involving exogenous ABA application has provided further insights into its role in dormancy regulation. Exogenous ABA treatment has been shown to induce dormancy and inhibit budbreak in several woody species [29,30]. On the other hand, research involving exogenous ABA application has provided further insights into its role in dormancy regulation. Exogenous ABA application has been shown to promote dormancy and suppress budbreak in many woody species [31]. This indicates that a regulatory mechanism not mediated by ABA may control the dormancy transition at certain phases or under particular conditions. Additionally, fluctuations in ABA levels are not always perfectly aligned with dormancy development. For example, in *Prunus avium*, ABA levels did not decrease until the buds reached ecodormancy [23,32,33]. Thus, whereas ABA is an essential regulator of dormancy, it seems that other regulatory networks, especially those responsive to environmental cues, may occasionally override ABA’s regulatory role.

## 4. Gibberellins (GAs) Positively Regulate Bud Dormancy

GAs are a family of tetracyclic diterpenoid compounds that are involved in a wide range of critical activities in the biological processes that occur in plants. On the other hand, only a small portion of the numerous GAs that have been discovered are indeed bioactive. These include GA_1_, GA_3_, GA_4_, and GA_7_, with GA_3_ being the most generally known form of gibberellic acid [34]. GAs stimulate both vegetative and reproductive growth by regulating processes such as floral development, sex expression, leaf shape, seed germination, stem elongation, and dormancy. It is widely accepted that GAs are critical in regulating bud dormancy, as substantial variations in bioactive GA levels are observed before and after dormancy [20]. Specifically, GA levels typically decrease during dormancy induction and rise during dormancy release and bud burst. These fluctuations in GA content have been documented in various woody species, including *P. avium* [21], hybrid aspen (*Populus tremula × P*. *tremuloides*) [35], *V. vinifera* [36], and *P*. *mume* [37].

At the onset of dormancy induction, a reduction in GA levels promotes growth inhibition and bud formation. Even under SD conditions, growth cessation can be partially reversed by exogenous GA application [38]. Interestingly, during long-day (LD) photoperiods, reducing GA biosynthesis in combination with low night temperatures failed to induce dormancy in hybrid aspen [38]. This indicates that GAs may play a function in the last stages of the SD photoperiodic pathways necessary for initiating dormancy. The role of GAs, namely GA_4_ and GA_3_, in the dormancy release of buds has been extensively documented across several species [36]. In *Populus*, for example, exogenous GA_4_ can replace the chilling requirement by upregulating genes sensitive to chilling (e.g., flowering locust T, FT) and triggering bud burst [35]. Recent studies also indicate that the impact of GAs on primordial growth and budbreak is limited once meristem activation occurs. Moreover, a preliminary increase in GA levels can inhibit budbreak, suggesting that GA plays distinct roles during the dormancy-to-growth transition [37].

Exogenous GAs influence dormancy release through several mechanisms. First, GAs modulate intercellular communication, which regulates the dormancy cycle. The progression of the dormancy cycle depends on the movement of macromolecules like FT, auxin, carbohydrates, and likely ABA [20], indicating that plasmodesmata permeability may be key in modulating dormancy. Recent research suggests that SD photoperiods can trigger ABA-mediated callose deposition and GA catabolism [39]. During prolonged dormancy, when GA concentrations are very low, the rate of material exchange in buds with surrounding tissues slows significantly [40]. GA_4_ may help restore plasmodesmata connectivity by inducing the production of β-1,3-glucanase, an enzyme that hydrolyzes callose [35]. Previous studies have also linked GA to ABA-mediated plasmodesmata closure through RNAi studies [39]. In this context, the removal of ABA reduces the synthesis of SHORT VEGETATIVE PHASE-LIKE (SVL) orthologous to the *Arabidopsis* SVP floral repressor. This, in turn, activates the expression of the GA catabolic gene *GA*_2_ *oxidase* and *CALS1*, leading to reduced GA levels. When exposed to SD photoperiods, high ABA levels inhibit *PKL* expression, promoting *SVL* activation, which drives GA catabolism and callose accumulation [39]. Furthermore, it has been shown that GAs stimulate the production of ROS, which are essential for the breaking of dormancy [36]. The sudden accumulation of ROS in the spring is highly connected with the budbreak that occurs in *V*. *vinifera* [41]. The production of ROS during the break of dormancy has been documented in several plant species, and it is believed to have a role in the break of buds [41,42,43]. Finally, GAs have the potential to trigger metabolic pathways that result in dormancy break. For instance, in *P*. *mume*, GA_4_ treatment enhances energy metabolic pathways, particularly those involved in sugar metabolism [44]. Soluble sugars are crucial for providing energy to buds as they exit dormancy, and sucrose may also act as a signaling molecule that enhances the expression of genes involved in cell division and the cell cycle [45].

## 5. Interaction Between GA and ABA During Bud Dormancy

ABA and GA levels are negatively associated during dormancy, and the ABA/GA ratio changes proportionately with dormancy duration [21]. Following dormancy, the fall in ABA level is dependent on a steady rise in GA level in Japanese apricot [46]. Experiments on transgenic plants revealed that changing one hormone might be responsible for modifying the metabolism of another. ABA pathway mutations increase GA concentration by encouraging GA biosynthesis genes like *GA3ox*, while GA reduction boosts ABA biosynthetic genes (*NCEDs* and *ABA1*) and decreases the ABA catabolic gene (*CYP707A*) [47,48]. These findings suggest that ABA and GA exert metabolic control over each other. According to recent transcriptome analysis, GA treatment decreases the expression of both ABA biosynthesis genes such as *NCED1*, *NCED1*, and *ZEP* as well as catabolic genes such as *CYP707A2* in the tea plant. The GA biosynthesis genes (*CsKO*, *CsKS*, *CsKAO*, *GA20ox2*, *GA20ox1*, and *GA3ox1*) were considerably upregulated by ABA treatment but the expression patterns of the catabolic gene *GA2oxs* were concentration dependent [49]. The relationship between GA and ABA also happens at the level of signal transduction. DELLA proteins are integral to the GA signaling pathway and function as a cross-talk node, integrating many signaling pathways of different hormones, including ABA [50,51]. DELLAs enhance the production of XERICO (a RING zinc finger protein), which has been shown to augment ABA synthesis in mutants exhibiting impaired GA signaling [52,53]. In tea plants, GA treatment enhances the expression of the negative mediator *PP2C* in ABA signaling while lowering the expression of the ABA receptor *PYL8e* [49]. Since stress-induced ABA may stabilize DELLA proteins, high ABA levels during dormancy induction might decrease GA responses [53]. Exogenous ABA hormone reduces GID1 expression in the GA receptor while enhancing DELLA family expression. Long-term ABA therapy, on the other hand, has been demonstrated to suppress DELLA expression [49].

## 6. Auxins Regulate Bud Dormancy

Auxin has been widely acknowledged for its role in promoting stem elongation while inhibiting the growth of lateral buds, a phenomenon referred to as apical dominance. Recent studies have also highlighted its involvement in regulating plant senescence, flowering processes, and responses to various stress factors [54,55]. IAA is perhaps the most common and extensively researched of the four naturally occurring auxins [56]. IAA, a potent growth stimulant, has been associated with the induction of dormancy release in many species. Preliminary studies indicated that auxin may expedite the degradation of dormancy callose in the phloem of *Magnolia kobus* and facilitate the restoration of symplastic pathways, essential for budbreak [57]. IAA concentrations in the tea plant (*Camellia sinensis*) remain low throughout the dormancy period and gradually increase after the emergence from hibernation until bud burst in the spring [58]. Moreover, free IAA content varies in the reverse direction of its conjugated form, indicating that conjugation may be an important mechanism in regulating endogenous IAA homeostasis during dormancy. Increments in IAA concentration after dormancy break have also been documented in Chinese fir (*Cunninghamia lanceolata*) and plum (*P. mume*) [59]. However, polarly transported IAA stimulates GA production, which is essential for development [60,61,62]. IAA might assist in dormancy break in cooperation with GA. Previous studies have demonstrated that IAA concentrations are primarily regulated through metabolic pathways, as evidenced by the significant expression of the IAA synthesis gene *YUC3* during natural dormancy release or in response to HC. Increased IAA levels, compared with controls, play a crucial role in initiating the cell cycle and activating dormant buds [63]. Transcriptomic studies indicated that the key components of the IAA signaling pathway expressed differently throughout the shift from dormancy to developing stage, and their possible functions remain unknown [33,59].

On the other hand, IAA seems to aggregate ambient inputs during dormancy induction. When strawberries were under SD photoperiod (subjected temperatures), IAA and transcript levels of polar auxin transport (*PAT*)-related genes (e.g., *PIN*) decreased significantly, which was followed by an elevation in global genomic DNA methylation and ABA levels [64]. When hybrid aspen is treated with SD photoperiod, PAT-related genes and auxin signaling repressor (AUX/IAAs) reduce auxin sensitivity, which is associated with the downregulation of activator AFRs and the upregulation of repressor ARFs [65]. According to transcriptome data, the majority of auxin-responsive genes in poplar and Japanese apricot are negatively regulated during dormancy [66,67]. Current findings revealed that the modification of auxin transport ability, auxin responsiveness, cellular auxin content, and conjugation may be included in the dormancy regulatory network.

## 7. Ethylene (ET) Regulates Bud Dormancy

ET has a broad range of impacts on several biological mechanisms including seed germination, blooming, senescence, and numerous stress responses [68]. ET’s role in dormancy is intimately linked to its production and signaling transmission pathway. ET biosynthesis begins with SAM synthetase converting methionine to S-adenoyl-methionine (SAM) [69]. ACC (1-aminocyclopropane-1-carboxylic acid) synthase converts SAM to ACC in the following rate-limiting and committed step (ACS). In the last stage, ACC oxidase (ACO) oxidizes ACC to generate ET, HCN, and CO_2_ [70]. ACO and ACS enzymes are major moderators of endogenous ET concentration in the mentioned biosynthetic pathway, but both are expressed by multi-gene families [71,72]. In *Arabidopsis*, five ET receptors have been discovered in the ET signaling pathway: *ERS1*, *ERS2*, *EIN4*, *ETR1*, and *ETR2* [70]. In the lack of ET, these receptors employ inhibit *EIN2* and *CTR1* (Constitutive Triple Response1), which is a membrane-spanning protein that acts as a regulatory element of ET signaling [73,74]. *EIN2* stagnates the downstream transcription factors (TFs) EIN3 (ETHYLENE INSENSITIVE 3)/EIL1 (EIN3-Line1) by provoking downregulation of F-box proteins [75]. In addition to the *EIN3* primary targets are the *ERF* gene family, which is part of the AP2/ERF superfamily and performs a significant role in adaptability to a variety of abiotic and biotic stimuli [76]. Evidence from several studies points to ET and its response pathway being engaged in dormancy control. Previous research suggested that ET levels are enhanced during both dormancy induction and bud dormancy release, and that the ET antagonist NBD (2,5-norbornadiene) promotes early dormancy break in micro potato tuber [77]. It was illustrated that an ET-insensitive mutation (*etr1-1*) results in the absence of terminal buds, reduced ABA production, and prolonged dormancy in *Betula pendula* (European white birch) under short-day photoperiod conditions [78]. Similarly, mutants with defective ET receptor gene (*DG-ERS1*) are unable to undergo dormancy at dormancy-provoking temperature in chrysanthemum [79]. Microarray and transcriptome investigations verified the role of ET in the development of dormancy, finding that the ET synthesis gene group and signaling component genes (like *ERF*, *ETR2*, *EIN3*, *EIN4*) in poplar are elevated under a dormancy-promoting environment [26,67]. ET may affect GA or the GA signaling pathway to promote dormancy, according to research on the interaction between ET and GA. It has been shown that active ET signaling triggers GA deactivation and DELLA deposition in response to environmental stress by increasing the expression of ET response factors (*ERF6*) [80,81]. Furthermore, previous studies revealed that ET may impact and alleviate DELLA through *CTR1* without the participation of GA [82]. When the association between phytochrome signaling and ET action was investigated, further evidence of the link between ET and GA was revealed [83]. This indicates that the principal signal transduction of phytochrome related to light responses may result in ET deposition and a decrease in GA during short days, ultimately leading to growth halt and the onset of dormancy.

ET was also mentioned as being involved in dormancy release. NBD, an inhibitor of ET signaling, increases ABA levels by breaking buds, indicating that ET is important for ABA breakdown and regulation of ABA signaling [19,28]. In *Arabidopsis*, ET inhibits *CBF* (C-repeat binding transcription factor, the cold tolerance gene) by activating *EIN3*, which inhibits *CBF* genes by binding to their promoters [84]. The following discoveries on grape bud data provide more support: (1) dormancy break stimuli such as sodium acid, heat shock, and HC may briefly stimulate ET production; (2) external ET treatment promotes budbreak; and (3) dormancy release is significantly postponed when ET signaling is prevented by *NBD* [28]. These results demonstrate that ET has various actions throughout bud dormancy, synergistically interacting with ABA during dormancy induction but interacting in an antagonistic manner after dormancy release. These results demonstrate that ET and its signaling pathway is required for dormancy formation, and that GA and ABA interact downstream of ET-mediated dormancy regulation. Previous studies demonstrated that increased levels of ROS cause budbreak, and ET is actively implicated within that mechanism. HC-induced gene expression differences demonstrated a link among dormancy release, hypoxia, oxidative stress, mitochondrial activity, signaling pathways, and ET production [28,41,85]. Plants produce ROS, such as H₂O₂, in response to HC, which triggers various pathways associated with dormancy release, particularly those involving antioxidant systems. Research has linked ET synthesis to heightened oxidative stress due to hydrogen cyanide production [85], with studies showing that hydrogen cyanide is more effective than ET in promoting bud burst [85]. ET may act as a mediator by activating the antioxidant system, such as catalase (CAT), to neutralize excess ROS [86]. Indeed, it has been argued that the buildup of ROS and their removal are critical phases in breaking dormancy [41,43].

## 8. Cytokinins’ (CTKs) Impact on Bud Dormancy

CTKs are a family of tiny molecules derived from adenine that perform essential roles in plant cellular activities such as apical dominance, cell division, differentiation, and stress tolerance [87,88]. CTKs are extremely crucial in regulating morphogenesis and meristem activity since their effects vary widely based on different cell and tissue type environmental variables and developmental stage. At the cellular level, CTKs can activate CDK (cyclin-dependent protein kinase) by dephosphorylating its tyrosine. The effect of CTKs is regarded to be essential for the proper advancement of the cell cycle, which would otherwise come to a stop at the G2 phase and be deemed insufficient [89]. Natural CTKs possess a varied array of side chains linked to the parent molecule adenine, and this structural variety facilitates remarkable sensitivity and specificity in the interactions between CTKs and receptors [90]. Adenosine phosphate-isopentenyltransferase (IPT) governs the rate-limiting step in CTK metabolism, whereas the primary catabolic pathway is CTK oxidation, which entails the elimination of side chains via the CTK oxidase/dehydrogenase1 enzyme. Although CTKs are highly transportable in plants and may travel long distances in xylem sap, locally generated CTKs have been proposed to be significant in dormancy management [91].

CTKs play significant roles in regulating dormancy cessation and the activation of latent buds from paradormancy (PD) [92]. Early studies revealed that CTK levels in xylem sap rise sharply in response to bud-bursting agents and remain elevated throughout the budburst process in apple plant [93]. Genetic studies suggest that LD photoperiods or HC treatment can increase CTK levels in grapevine cuttings by upregulating CTK biosynthesis genes, such as IPT and LOG1, while downregulating CTK catabolism genes like *CTKX*. Furthermore, temporal expression analyses indicate that stress-induced CTK elevation may enhance the expression of cell-cycle-related genes, thereby promoting cell division and cellular respiration—critical processes required for the activation of dormant buds [63]. Prior research has shown that elevated levels of CTK in IPT-expressing potato tubers facilitated an earlier onset of dormancy break; conversely, decreased CTK levels due to CTKX overexpression inhibited cellular metabolism and development, leading to an absence of response to GA_3_ and an extended dormancy duration [63]. Prior research has shown that elevated quantities of CTK in IPT-expressing potato tubers facilitated an earlier onset of dormancy break, but decreased CTK levels due to CTKX overexpression inhibited cellular metabolism and development, leading to an absence of response to GA_3_ and an extended dormancy duration [94]. Another indication of CTK’s role in dormancy release is its role in light signal mediation. Several His kinases have been discovered as CTK receptors, including *AHK4*, *AHK3*, and *AHK2*, which effectively govern CTK signals by phosphorylating His-containing phosphor transfer proteins [95,96,97]. Under CTK accumulation, these CTK receptors adversely influence ABA responses using a loss-of-function strategy [98]. However, light can modulate CTK signaling by regulating the transcription of these CTK receptors [99]. This observation was corroborated by the discovery, in *Rosa hybrida*, that CTK participates in the earliest actions of the light signaling system that encourages bud development [100]. These findings imply that light-mediated increases in CTKs in dormancy release may contribute to ABA reduction. These findings indicate that CTK is a crucial regulator of dormancy release and that CTK promotes meristematic activity by acting upstream of the ABA and GA response pathways.

## 9. Jasmonates (JAs) Regulate Bud Dormancy

JAs are lipid-based plant hormones that govern a variety of mechanisms in plant growth and defense, including dormancy [101]. JAs, as well as their metabolic precursor conjugate form JA-Ile and 12-oxophytodienoic acid (OPDA), have all been shown to be effective signaling molecules. JAs have a prominent impact on bud dormancy. After bud burst, JA levels in *Fagus sylvatica* (beech trees) increased considerably [102]. According to the same, as the buds move from dormancy to vigorous sprout, the concentration of JA-Ile in potato tubers continuously rises [102,103]. Transcriptomic studies have shown that the JA pathway remains suppressed during dormancy but becomes active during the endodormancy and budbreak stages [40]. These findings suggest that JAs play a role beyond merely inhibiting growth, contributing to the processes involved in dormancy release and bud activation. Prior research has demonstrated that the JA signaling pathway is active during the cold adaptation phase, which is strongly associated with dormancy. JA ZIM-DOMAIN proteins are essential JA signaling repressors and may form a JA receptor complex with CORONATINE INSENSITIVE1 (F-box protein COI1) [104,105]. JAZ (jasmonate ZIM-domain) proteins are associated proteins degradation when JA attaches to the complex, releasing multiple groups of TFs, including the helix–loop–helix MYC4, MYC2, and MYC3 [106]. MYC2 (critical transcriptional stimulator of JA responses) has been shown to stimulate *Arabidopsis* gene *SAG29* (*SENESCENCE-ASSOCIATED GENE29*) through integrating to its promoter as well as encouraging JA persuaded by leaf senescence, whereas other bHLH TFs (bHLH13, bHLH14, bHLH03 and bHLH17) can attach to *SAG29* promoter to mitigate the important influence of *MYC2/MYC3* [107]. The cold response system is well understood via *ICE* (inducer of CBF expression), CBF-TFs, and numerous *COR*s (cold-regulated genes) [108,109]. More data have recently emerged that demonstrated a direct relationship among ICE-CBF-COR signaling cascade and JA signaling. First, yeast two-hybrid investigations showed links between ICE proteins, JA signaling repressors, and JA-induced JAZ protein degradation, which resulted in *COR* gene activation [110]. Furthermore, it has been demonstrated that *MYC2*, a transcriptional regulator induced by JA, engages with *ICE1* and enhances the cold response network. This supports the notion that JA serves as a significant signal upstream in the ICE-CBF-COR pathway that enhances cold tolerance [111]. Third, it was observed that exogenous JA injection increased *Arabidopsis* plant chilling tolerance and cold exposure stimulated JA production [110]. However, it has to be shown that cross talk among cold-responsive and JA pathways occurs during dormancy, particularly in woody plants. Interactions between hormonal signaling components (such as *EIN3/EIL1* and *DELLA*) and JAZ proteins suggested that JA may be broadly and intimately linked to the regulatory network of acclimatization and dormancy, and by investigating its interactions with other hormones, its function will be completely understood [112,113].

## 10. ROS Impact on Bud Dormancy

Hydrogen peroxide (H_2_O_2_) and superoxide (O_2_^−^) are essential components in redox signal transmission at quantities below the deadly threshold [114,115]. Non-enzymatic and enzymatic antioxidants that maintain sub-lethal levels of ROS in cells are capable of redox reactions and ROS turnover. Endodormancy break and bud burst are often associated with higher H_2_O_2_ and O_2_^−^ levels in the buds [116]. Adequate chilling exposure enhances H_2_O_2_ concentration of floral buds with the initiation of endodormancy emission in Japanese pear, but inadequate chilling exposure does not [117]. In addition, a budbreak agent, HC, promotes a temporary upregulation of H_2_O_2_ in grapes, resulting in dormancy release [118,119]. O_2_^−^ is produced by NADPH oxidase, a plasma-membrane-based enzyme that converts electrons from cytoplasmic NADPH to oxygen. NADPH oxidase has been shown to stimulate the breaking of dormancy in *Arabidopsis* seeds and potato tubers [42,120]. Furthermore, NADPH oxidase blockers reduce seed germination in barley [121] and tuber sprouting in potatoes [42]. However, the involvement of NADPH oxidase in the transfer to dormancy in deciduous fruit trees is completely unknown. Glutathione (non-enzymatic antioxidants), superoxide dismutase (SOD) (enzymatic antioxidants), CAT, glutathione peroxidase, and glutathione reductase perform critical roles in ensuring appropriate ROS levels during dormancy [122,123]. Several transcriptome investigations revealed the upregulation of the same set of genes following exposure to cooling, H_2_O_2_, or HC. Glutathione reductase, glutathione-s-transferase, CAT, SOD, ascorbate peroxidase, and redox-related genes are among those that are involved [28,124].

## 11. *DAM*/*SVP* Genes and Related Transcription Factors Associated with Bud Dormancy

Over the past decade, significant research efforts have been dedicated to uncovering the genetic mechanisms underlying bud dormancy. Among the identified regulators, a specific group of transcription factor (TF) genes has garnered attention across various plant taxa. These include the *DORMANCY-ASSOCIATED MADS-box* (*DAM*) genes in Rosaceae fruit trees, *SVL* genes in *Populus*, and *SHORT VEGETATIVE PHASE 2 (SVP2)* genes in kiwifruit (*Actinidia* spp.) [39,125,126]. These genes are evolutionarily linked to the *SVP* and *AGAMOUS-LIKE 24* (*AGL24*) genes, which are key MADS-box regulators involved in floral development and transition in *Arabidopsis* [127]. *DAM* genes were first identified in the ever-growing (*evg*) mutant peach [128], which exhibits continuous growth without responding to environmental signals, bypassing growth cessation [129]. Genetic studies in Rosaceae fruit trees including, *P. avium* [130], *Malus domestica* [131], *P. mume* [132], and *Pyrus communis* [133] have pinpointed quantitative trait loci (QTLs) co-localizing with *DAM* gene regions, strongly suggesting their involvement in dormancy regulation. A recent genome-wide association study (GWAS) in peach further confirmed the role of *PpDAM6*, which overlaps with the EVG locus and functions similarly to SVL in hybrid aspen. *PpDAM6* is a positive regulator of dormancy, and its loss of function reduces the chilling requirement for dormancy release, though it does not eliminate it [134]. In contrast, transgenic apple plants with reduced expression of *MdDAM1* and *MdDAM4* fail to form terminal buds, resulting in the absence of dormancy induction [135]. Beyond Rosaceae species, *DAM*/*SVP* genes have been identified in kiwifruit, where genetic and physiological evidence indicates that *SVP2* helps prevent premature budbreak in axillary buds during dormancy [136]. Consistent with their dormancy-regulating roles, *DAM*/*SVP* genes exhibit distinct seasonal expression patterns, with peak expression observed during dormancy and repression triggered by chilling temperatures [126]. These seasonal expression profiles are notably consistent across species, underscoring their conserved function in dormancy and budbreak regulation.

TFs played an important role in gene expression, which was mostly regulated via connections with chromatin-modifying machinery and promoters [137]. TFs play a key role in bud dormancy regulation. *bZIP67* (Lucine zipper 67) functions downstream of LEC1 (Leafy cotyledon 1) to transactivate DOG1 by binding G-box-like *cis* sites on the DOG1 promoter, promoting primary dormancy [138]. Members of the TF family such as C3H, MYB, WRKY, HD-ZIP, NAC, and AP2/ERF were found in both induced and ABA-repressed DEGs, demonstrating that numerous members of the same TFs family may work adversely to control germination and dormancy. Many indications have recently showed that these TFs play critical roles in dormancy control. WRKY41 is an ABI3 positive regulator that promotes primary seed dormancy. The ABA sensitivity of wrky41 mutants was similar to that of the abi3 mutation [139]. MYB96 promotes ABA synthesis by favorably regulating NCEDs, while negatively regulating *GA20ox1* and *GA3ox1* to lower GA content during the germination phase [140]. In *Arabidopsis* and rice, the OsAP2-39 type TFs ABI4 and AP2/ERF were shown to be engaged in ABA-GA antagonistic cross talk [141]. Furthermore, MapMan analysis of Affymetrix tiling array data from the complete genome comparing ABA metabolism mutants and wild-type revealed that *MYB*, *MADS-box*, *WRKY,* and *NAC* genes may be involved in dormancy control [142,143].

## 12. Artificial Dormancy Mitigation

### 12.1. Plant Growth Regulators

The use of plant growth regulators (PGRs) is an appropriate choice that reduces the chilling requirement or stimulates the induction of the buds into dormancy, resulting in enhanced budbreak in the spring. GAs are particularly significant as they are believed to contribute to growth resumption after dormancy release. Numerous studies on fruit trees have underscored their involvement in regulating dormancy progression. However, direct measurements of GAs are relatively rare, with most research emphasizing their metabolic pathways and the effects of exogenous applications. Additionally, GAs have shown potential as a substitute for chilling requirements in certain contexts [144,145], and GAs production is endorsed by dormancy breaking reagents [146]. However, the maximum concentrations of GA_3_ and GA_1_ were observed during endodormancy emission in dormant buds and afterwards reduced [46,147]. Overall, expression studies confirm this, with upregulation of GA20-oxidase and GA3-oxidase, which are essential for bioactive GA production, after chilling treatment [35], even around dormancy break [148]. GA2-oxidase (*GA2ox*) genes, encoding enzymes necessary for the inactivation of bioactive GA_1_ and GA_4_, are elevated in dormancy and dormancy break in Japanese apricot buds, but also during ecodormancy in Japanese pear buds [149]. Thus, the control of GAs seems to be tightly controlled between synthesis and degradation, and they may increase growth rate under favorable conditions.

### 12.2. Nanotechnology

Winter’s cold temperatures can make it harder for plants to wake up from their dormant state. The effect of chilling builds up over time, and the buds are fully awakened when they reach a certain point. After a certain amount of heat builds up above a certain base temperature, the buds open, and growth starts again in the spring. As a consequence of global warming, the chilling need may not be entirely satisfied, causing bud burst to be delayed; conversely, bud burst may occur earlier than usual in an environment where the chilling requirement is now significantly surpassed. To compensate for the absence of winter cold needed by deciduous fruit trees grown in mild winter climates, several initiatives have been devoted to break up bud dormancy utilizing natural and synthetic growth agents [43]. PGRs utilization is an appropriate approach that is now used. PGRs play vital roles in equal early bud burst in plants grown in northern areas. Early budbreak led to early fruiting in market, which has a high return for the growers. However, to achieve an equal budbreak, several PGR spray treatments are used, which results in phytotoxicity for human health. Residues of PGRs in agricultural products are a significant concern due to their potential health risks. Research has linked these residues to a wide range of adverse health effects, including hepatotoxicity (damage to liver cells), nephrotoxicity (kidney impairment), genotoxicity (DNA damage that can lead to mutations), and neurotoxicity (harm to the nervous system). Additionally, prolonged exposure has been associated with more severe conditions such as carcinogenicity (cancer development) and teratogenicity (birth defects) [150]. There is an urgent requirement to discover an approach that will not only assist in cutting down on the PGRs dose treatment but will also prove to be quite effective against even budbreaking. Additionally, the approach needs to be environmentally friendly.

Nanotechnology is one of the potential techniques that may be used in agriculture and forestry to reduce the influence of endogenous hormones and help plants to grow better in adverse climates. In the year 1974, Norio Taniguichi, a professor at Tokyo University of Science, is credited as being the first person to use the word “nanotechnology” [151]. Although, the word “nanotechnology” has long been used in a range of aspects, the concept that nanoparticles (NPs) may be useful in agriculture expansion is a relatively new technical invention, which is still in the early stages of development [152]. Recent advances in the production of nano materials of various shapes and sizes have resulted in a diverse range of applications in medical, agriculture, environmental research, and food processing. Agriculture has always befitted from technological advancements throughout history. In addition, since agriculture confronts many and unexpected issues, such as decreased crop production owing to abiotic and biotic challenges such as nutrient insufficiency and pollution, the introduction of nanotechnology has presented intriguing applications for precision agriculture. Notable scientific discoveries include recent developments in tissue engineering and engineered nanomaterials-based controlled distribution of CRISPR/Cas (CRISPR-associated protein) mRNA and sgRNA for crop GM (genetic modification) [153,154,155]. Nanotechnology provides promising solutions to an expanding array of environmental challenges. For instance, the development of nanosensors holds significant potential for detecting environmental stress and enhancing plant resistance to diseases [144,145]. These innovations underscore the transformative role of nanotechnology in addressing critical issues. By focusing on problem identification and fostering collaborative approaches, advancements in this field can contribute significantly to sustainable agricultural development [156,157]. Such progress not only supports environmental sustainability but also offers substantial societal benefits, promoting equity and resilience on a global scale.

## 13. Environmental Challenges

Climate change is widely recognized as one of the most pressing environmental challenges of our time. The Intergovernmental Panel on Climate Change (IPCC) reports that the global average surface temperature rose by 0.74 °C over the past century. Projections indicate that this warming trend will continue, with temperatures expected to increase by an additional 1.1 °C to 6.0 °C by the end of the current century, depending on greenhouse gas emissions and mitigation efforts [158]. This escalation poses significant risks to ecosystems, agriculture, and human societies worldwide [15,159]. This threat is closely associated with the inability of plants to adapt their dormancy and growth cycles to future climatic conditions. Key factors contributing to this issue include the increased frequency of spring frosts and insufficient fulfillment of chilling requirements. These disruptions can severely impact plant development, yield, and overall agricultural productivity in a changing climate [160]. The dormancy and growth cycle in plants is linked with environmental and climatic circumstances. Temperate tree species have evolved a technique based on bud dormancy to adjust to alternating well-differentiated seasons, which serves to preserve the bud from winter cold, ensuring that blooming happens under favorable circumstances. A physiological categorization system had been established for the various dormancy stages that considers the inputs and sources of dormancy-imposing stimuli [5]. Ecodormancy is a growth inhibition caused by cold temperatures that occurs in early spring and late winter. Endodormancy is defined by severe dormancy, which is like correlative inhibition or apical dominance. In temperate fruit species, blooming requires exposure to cold in winter (meeting chilling requirements for breaking endodormancy) followed by a warm phase (meeting heat requirements). Thus, phonological features like chilling hour needs influence blooming date and are critical for synchronizing flowering time with environment conditions. As a result, winter and spring climatic warming is to blame for various disturbances that have already emerged in temperate fruit plants [15,159]. Numerous species have exhibited earlier budbreak and flowering dates, a phenomenon that heightens their vulnerability to frost damage [161]. Frost damage poses a particularly significant risk, as it results in greater economic losses than any other weather-related event, impacting crop yields and agricultural revenues globally [162]. In addition, climate change will result in an uncompleted dormancy break in various subtropical and temperate regions [15], which may lead to a delay in bud burst, a poor budbreak%, and an absence of leaf formation and bloom uniformity [163]. Such changes are likely to result in increased flower-bud loss and the development of morphological abnormalities, which could severely impact fruit yield. This issue is particularly critical for species with limited availability of commercially viable low-chill cultivars, where the lack of adaptation to low-chill conditions exacerbates the risk of reduced productivity.

## 14. Conclusions

Dormancy serves as a crucial adaptive mechanism for temperate and boreal plants, especially deciduous fruit trees, allowing them to endure winter conditions. This review examines the regulatory mechanisms of dormancy, emphasizing the influence of environmental factors and plant hormones such as ABA, GAs, and CTKs. These hormones regulate the initiation, progression, and release of dormancy, enabling plants to align their growth with seasonal variations. Recent developments in molecular biology and proteomics have enhanced our comprehension of the regulatory mechanisms governing cellular dormancy in response to genetic and environmental signals. Climate change presents considerable challenges, likely disrupting dormancy cycles and influencing phenological events such as flowering and fruiting. This highlights the necessity for adaptive strategies, including the selection of climate-resilient cultivars and the development of treatments to adjust dormancy timing. Research into the molecular, genetic, and hormonal regulation of dormancy is essential for optimizing agricultural practices and ensuring the sustainability of fruit production in changing climates.
